# Fluctuations in Blood Marginal Zone B-Cell Frequencies May Reflect Migratory Patterns Associated with HIV-1 Disease Progression Status

**DOI:** 10.1371/journal.pone.0155868

**Published:** 2016-05-20

**Authors:** Julie Gauvin, Josiane Chagnon-Choquet, Johanne Poudrier, Michel Roger, Montreal Primary HIV Infection and Slow Progressor Cohorts

**Affiliations:** 1 Laboratoire d’immunogénétique, Centre de Recherche du Centre Hospitalier de l’Université de Montréal (CRCHUM), Montréal, Canada; 2 Département de Microbiologie, Infectiologie et Immunologie de l‘Université de Montréal, Montréal, Canada; University of Cape Town, SOUTH AFRICA

## Abstract

We have previously shown that overexpression of BLyS/BAFF was associated with increased relative frequencies of innate “precursor” marginal zone (MZ)-like B-cells in the blood of HIV-1-infected rapid and classic progressors. However, along with relatively normal BLyS/BAFF expression levels, these cells remain unaltered in elite-controllers (EC), rather, percentages of more mature MZ-like B-cells are decreased in the blood of these individuals. Fluctuations in frequencies of blood MZ-like B-cell populations may reflect migratory patterns associated with disease progression status, suggesting an important role for these cells in HIV-1 pathogenesis. We have therefore longitudinally measured plasma levels of B-tropic chemokines by ELISA-based technology as well as their ligands by flow-cytometry on blood B-cell populations of HIV-1-infected individuals with different rates of disease progression and uninfected controls. Migration potential of B-cell populations from these individuals were determined by chemotaxis assays. We found important modulations of CXCL13-CXCR5, CXCL12-CXCR4/CXCR7, CCL20-CCR6 and CCL25-CCR9 chemokine-axes and increased cell migration patterns in HIV progressors. Interestingly, frequencies of CCR6 expressing cells were significantly elevated within the precursor MZ-like population, consistent with increased migration in response to CCL20. Although we found little modulation of chemokine-axes in EC, cell migration was greater than that observed for uninfected controls, especially for MZ-like B-cells. Overall the immune response against HIV-1 may involve recruitment of MZ-like B-cells to peripheral sites. Moreover, our findings suggest that “regulated” attraction of these cells in a preserved BLyS/BAFF non-inflammatory environment, such as encountered in EC could be beneficial to the battle and even control of HIV.

## Introduction

Promising vaccine strategies as well as studies with individuals presenting natural immunity have highlighted the importance of B-cells in the immune response against HIV [1]. These are likely involving orchestration of first-line innate immunity in conjunction with matured high affinity adaptive responses, and expected to operate at peripheral mucosal sites, which are ports of entry and replication for the virus. Understanding the nature and how B-cell populations are recruited and maintained within peripheral and mucosal niche [2,3] to facilitate or control HIV disease progression is important to the design of effective preventive/therapeutic approaches.

The B-cell compartment is impeded in the majority of HIV-infected individuals early on, throughout the infection, and not fully restored by therapy [4,5]. Despite a reduction in total B-cells, we have observed augmented frequencies of a population presenting features shared by both transitional immature (TI) and innate marginal zone (MZ) B-cells, designated as “precursor” MZ-like, in the blood of HIV-1-infected rapid and classic progressors [6,7]. Importantly, these were concomitant with high levels of BLyS/BAFF in plasma and on the surface of blood mDCs in these individuals, as soon as in the acute phase and persisted throughout infection despite highly active therapy. Most importantly, in aviremic slow progressors also referred as elite-controllers (EC), BLyS/BAFF levels were preserved and precursor MZ-like B-cell frequencies remained unaltered. Rather, we found that percentages of MZ-like B-cells presenting a more “mature” profile were decreased when compared to rapid and classic progressors, as well as HIV-negative individuals. These findings are in line with growing evidence suggesting that innate B-cell responses are involved in the fight against HIV [8].

In an effort to further understand the differences in blood B-cell population dynamics associated with disease progression vs control, we have assessed chemokine-ligand(s) axes presenting B-cell tropic potential towards peripheral lymphoid and mucosal sites namely CXCL13-CXCR5, CXCL12-CXCR4/CXCR7, CCL20-CCR6 and CCL25-CCR9 [9].

## Methods

### Subjects

Thirty-one individuals from the Montreal Primary HIV-1-Infection cohort were selected and divided into 13 rapid and 18 classic progressors based on their blood CD4^+^ T-cell counts. The date of infection was estimated using criteria established by the Acute HIV-Infection and Early Disease Research Program (NIAID, Bethesda, MD). Rapid progressors had blood CD4^+^ T-cell counts below 250 cells/mm^3^ within 2 years of infection. Blood samples were taken in acute (0–3 months) and/or early (5–8 months) phases of infection, and 3–6 and 9–12 months after initiation of antiretroviral therapy (ART). Classic progressors were ART-naive individuals whose blood CD4^+^ T-cell counts remained above 500 cells/mm^3^ for the 2 year follow-up. Blood samples were obtained in the acute, early and chronic (24 months) phases of infection. Blood samples from 12 slow progressors (6 viremic: low detectable viral load, and 6 aviremic: undetectable viral load) were obtained from the Montreal Slow Progressors cohort. These are patients with CD4^+^ T-cell counts that remain above 500 cells/mm^3^ after being infected for 8 years or more. Lastly, blood samples were obtained from 17 age- and sex-matched HIV-negative controls. Written informed consent was obtained from all subjects, and research conformed to guidelines and was approved by the CRCHUM Ethics Review board (project #SL05.028).

HIV plasma viral loads were determined with the Versant HIV-1 RNA 3.0 Assay (Siemens Medical Solutions Diagnostics, Tarrytown, NY). Blood CD4^+^ T-cell counts were assessed as reported [10]. The subjects did not present co-infections with syphilis and hepatitis B or C.

### Chemokine receptor expression by blood B-cells

Cryopreserved peripheral blood mononuclear cells (PBMCs) were processed for multi-color flow-cytometry as reported [6,7]. We used Aqua-LIVE/DEAD exclusion Fixable Stain (Invitrogen/Life technologies, Eugene, OR). The following conjugated mouse anti-human monoclonal antibodies were used: PacificBlue-anti-CD19, APC-Cy7-anti-CD10, AlexaFluor647-anti-CCR9 (BioLegend, San Diego, CA); AlexaFluor700-anti-CD27, FITC-anti-IgM, PE-anti-CD21, APC-anti-CXCR4 (BD-Biosciences); PerCP-eFluor710-anti-CD1c, PE-Cy7-anti-CCR6 (eBioscience, San-Diego, CA); APC-anti-CXCR7 (LifeSpan BioSciences, Seattle, WA). We have also used a biotinylated rat anti-human-CXCR5 (BD-Biosciences). Cells were kept at 4°C in 1.25% paraformaldehyde overnight prior to analysis. Data acquisition of 10^5^ live PBMC events per sample was performed with LSRFortessa (BD-Biosciences), and analysis was done with FlowJo7.6.3 software (TreeStar, Ashland, OR). All stainings were compared to that of fluorescence minus one (FMO) values and isotype controls. Anti-mouse Ig(κ) and Anti-Rat Ig(κ) Compbeads (BD-Biosciences) were used to optimize fluorescence compensation settings. CS&T beads (BD) were routinely used to calibrate the LSRFortessa to exclude the possibility of instrument-related fluorescence intensity changes over time, and we verified consistency prior to each data acquisition session using application settings based on Rainbow beads (BD).

### Plasma chemokine concentrations

Levels of CXCL11 (RayBiotech, GA), CCL25 and CCL20 (R&D Systems, MN) were determined using commercial ELISA Kits. Levels of CXCL12 and CXCL13 were measured using the Milliplex Human Cytokine/Chemokine Kit (Millipore, MA).

### Chemotaxis assays

Chemotaxis assays were performed as described [11]. Briefly, B-cells were negatively enriched from PBMCs of subjects randomly selected from the HIV-uninfected, HIV-infected classic progressor and aviremic slow progressor/EC groups using immunomagnetic-based technology (Dynabeads, Dynal-Invitrogen/Life Technologies). CD19+, CD14-, CD56-, CD3- and CD11c- staining ensured >95% purity of B-cells. 1.5x10^5^ B-cells were allowed to migrate through 5μm pore-size filters of transwell inserts (Corning, NY) for 3 hours at 37°C in response to either of medium alone or human recombinant CXCL13 (500ng/ml) [11], CXCL12 (250 ng/ml) [11], CCL20 (100 ng/ml) [12] and CCL25 (100 ng/ml) [13] (RayBiothech). Due to sample limitations, we used fixed chemokine concentrations as described [11–13]. Migrated cells were collected, stained and counted by flow-cytometry for 2 minutes. B-cell populations were analyzed as stated above. Wilcoxon paired T-test showed significant difference (p-value = 0.0033) in the viability of migrated (64.6%) and un-migrated (56.4%) B-cells after 3 hours, invariably of chemokine exposure. Migration is presented as an index of migrated cells towards each chemokine divided by the spontaneous migration of each sample, as described [14].

### Statistical analyses

Statistical significance of differences between groups was assessed with Fisher exact test for categorical variables and unpaired Student *t*-test when continuous variables were normally distributed or with Mann-Whitney U test otherwise. Wilcoxon signed-rank test was used for pairwise comparisons of different phases of infection within each group. Corrections for multiple comparisons were applied for comparisons across time for each groups of HIV infected individuals. Analyses were performed using GraphPad Prism 5.03 for Windows (GraphPad Software Inc, La Jolla, CA).

## Results

**Fig 1:**
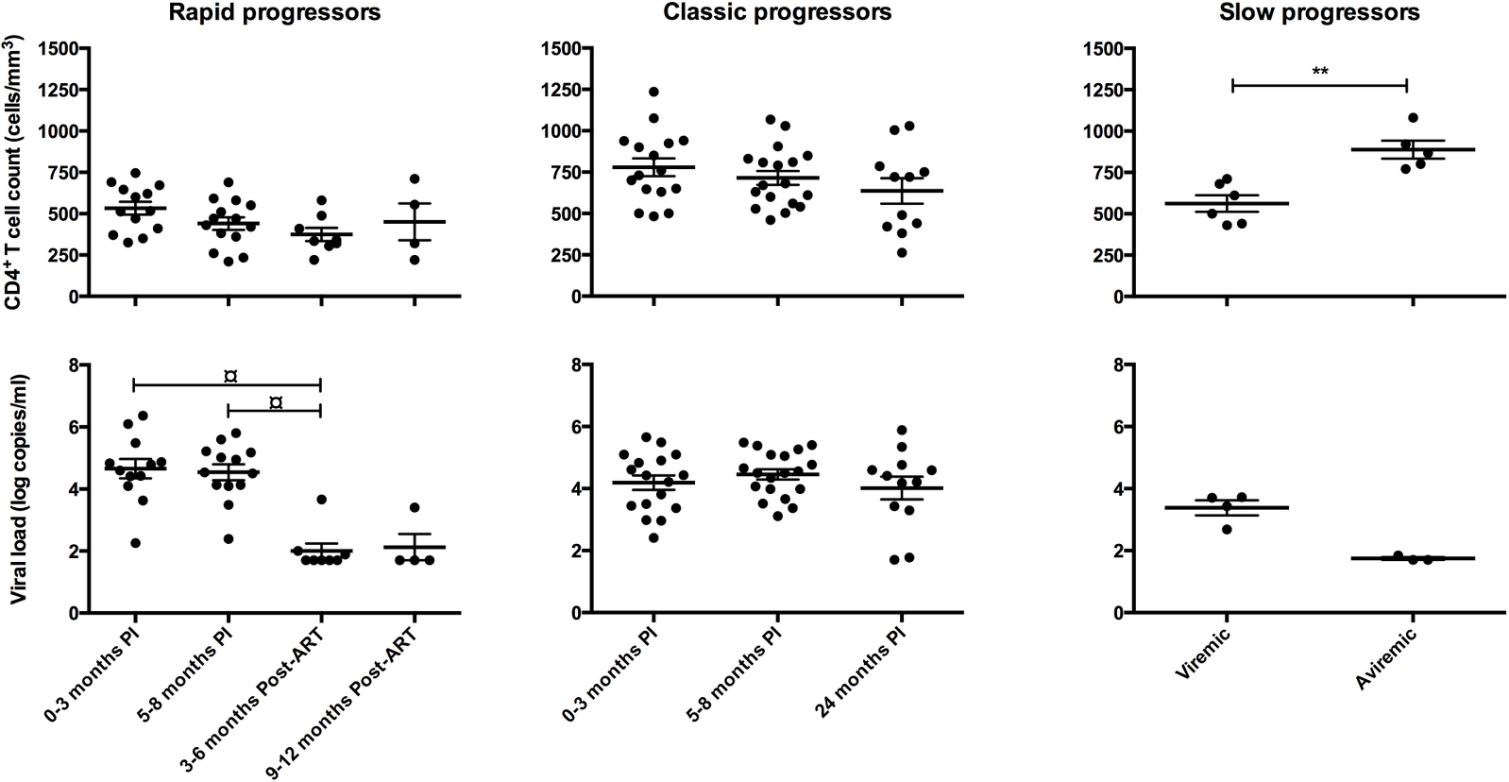
Longitudinal variations of blood CD4+ T-cell counts and viral loads in HIV-1 infected individuals. (A) Blood CD4+ T-cell counts (cell/mm3) were determined by flow-cytometry in rapid progressors (left panel; 0–3 months PI (n = 13), 5–8 months PI (n = 14), 3–6 months ART (n = 8), 9–12 months ART (n = 4)), classic progressors (middle panel; 0–3 months PI (n = 16), 5–8 months PI (n = 18), 24 months PI (n = 11)), and slow progressors (right panel; viremic (n = 6), aviremic (n = 5)). (B) Viral loads (log copies/ml) were quantified by in vitro signal amplification nucleic acid probe assay of HIV-1 RNA (bDNA) in the plasma of rapid progressors (left panel; 0–3 months PI (n = 12), 5–8 months PI (n = 13), 3–6 months ART (n = 8), 9–12 months ART (n = 4)), classic progressors (middle panel; 0–3 months PI (n = 17), 5–8 months PI (n = 19), 24 months PI (n = 12)), and slow progressors (right panel; viremic (n = 4), aviremic (n = 3)). The same values for HIV-negative donors (n = 18) in the left, middle and right panels are used as a control group. Cell populations and viral loads were compared using the Wilcoxon signed-rank test and Mann-Whitney U test for pairwise comparisons of different phases of infection within each group and between the study groups, respectively. Data shown are mean ±SEM. Significance levels are shown as ** p<0.001; ¤ p<0.05 for pairwise comparisons. PI, post-infection; ART, antiretroviral therapy.

Socio-demographic and clinical characteristics of HIV-1-infected individuals are shown in Table 1, and longitudinal assessment of blood CD4^+^ T-cell counts and viral loads are depicted in Fig 1. There were no significant correlations between blood CD4^+^ T-cell counts or viral loads and B-cell populations, chemokine plasma levels or chemokine receptor expression, either within groups or among all subjects during early or chronic infection (data not shown).

**Table 1:** Sociodemographic and clinical characteristics of HIV-1 infected individuals.

	Rapid progressors	Classic progressors	Viremic Slow Progressors	Aviremic Slow Progressors	*p*
**Characteristics**	(n = 13)	(n = 17)	(n = 7)	(n = 6)	
Age at first visit	34 ± 7	38 ± 8	46 ± 7	44 ± 11	0.005^1^
Sex (male/female)	12/1	17/0	7/0	3/3	0.0025
Race (Caucasian/other)	12/2	16/1	6/1	5/1	ns
Route of transmission	8 MSM, 3 HS, 1IDU	16 MSM, 1 IDU	7 MSM	3 MSM 3 HS	0.045
**CD4**^**+**^ **T cell count (cells/mm**^**3**^**)**					
Acute phase	533 ± 140	781 ± 213	na	na	0.002
Early phase	446 ± 146	714 ± 186	na	na	0.0008
Chronic phase	400 ± 152	629 ± 244	562 ± 122	888 ± 122	0.011^2^
Nadir	255 ± 118	431 ± 141	506 ± 129	506 ± 175	0.0005^3^
**Viral load (x 10**^**3**^**)**					
Acute phase	366 ± 705	76.2 ± 126	na	na	NS
Early phase	121 ± 186	71.2 ± 108	na	na	NS
Chronic phase	7.65 ± 15	37.7 ± 62	3.01 ± 1.9	<0.05^4^	0.003^5^
Peak	570 ± 808	202 ± 236	8.47 ± 6.46	0.06 ± 0.03	0.0001^6^

Age, CD4 and viremia are expressed as mean ± SD. Sex, race and route of transmission were compared using Fisher exact test. Pairwise comparisons of CD4 and viremia for early phases were performed using unpaired Student’s *t* tests. Comparisons among all groups (age at first visit, CD4 and viremia in the chronic phase and nadir CD4) were performed with the one-way analysis of variance test. MSM, men who have sex with men, HS, heterosexuals, IDU, intravenous drug users; n, numbers; NS, not significant; na, not available.

^1^ P = 0.004 and 0.05 for the comparison of age between rapid and viremic slow progressors, and classic and viremic slow progressors, respectively, as determined by the Mann-Whitney test.

^2^ P = 0.004 for the comparison of CD4^+^ T cells/mm^3^ in chronic phase between rapid progressors and aviremic slow progressors as determined by the Mann-Whitney test.

^3^ P = 0.0008 and 0.001 and 0.02 nadir CD4 for the comparison between rapid and classic progressors, rapid and viremic slow progressors, and rapid and aviremic slow progressors, respectively, as determined by the Mann-Whitney test.

^4^ 50 copies/ml corresponds to the detection threshold of the viral load test.

^5^ P = 0.002 for the comparison of viremia in chronic phase between classic progressors and aviremic slow progressors as well as for rapid and aviremic slow progressors, as determined by the Mann-Whitney test.

^6^ P = 0.006, 0.0007, 0.0005, 0.0004 and 0.001 for the comparison of peak viremia between rapid progressors and viremic slow progressors, rapid progressors and aviremic slow progressors, classic progressors and viremic slow progressors, classic progressors and aviremic slow progressors, and viremic and aviremic slow progressors, respectively, as determined by the Mann-Whitney test.

### Longitudinal measurements of CXCL13 plasma levels and CXCR5 expression by blood B-cells of HIV-1-infected individuals with different rates of disease progression

**Fig 2:**
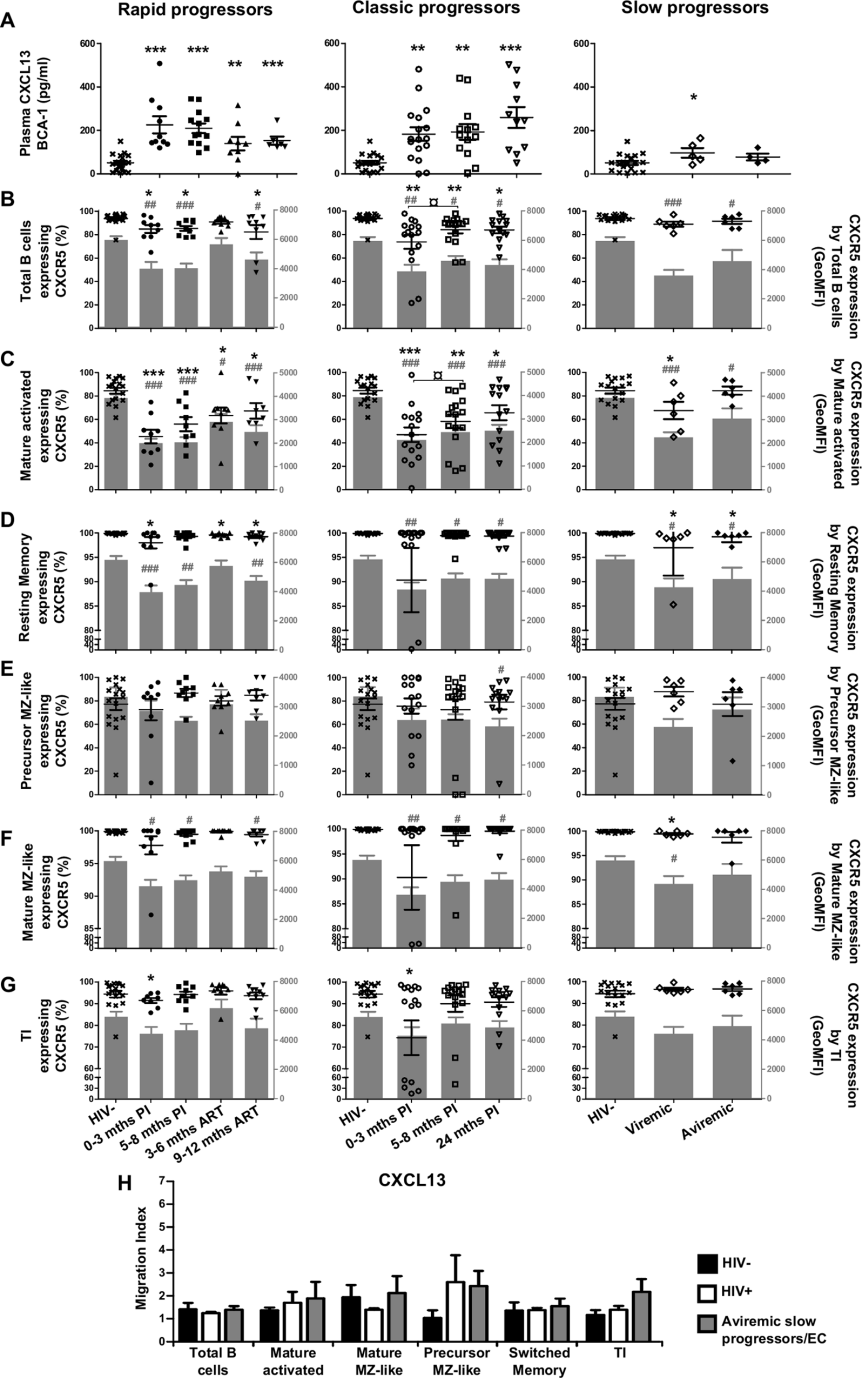
Analysis of plasma CXCL13 levels, CXCR5 expression and migratory potential by blood B-cells of HIV-infected individuals. (A) Plasma concentrations (pg/ml) of CXCL13 in rapid (left panel), classic (middle panel) and slow progressors (right panel). The same HIV-negative values are used as a control for all three panels. Frequencies of B-cells expressing CXCR5 (left y axis) and levels of CXCR5 surface expression (geometric mean fluorescence intensity—geoMFI) (right y axis) by (B) total, (C) mature activated, (D) resting switched memory, (E) ‘precursor’ marginal zone (MZ)-like, (F) ‘mature’ MZ-like and (G) transitional immature (TI) B-cells of rapid (left panel), classic (middle panel) and slow progressors (right panel). The same HIV-negative values are used as a control for all three panels. Data are expressed as percentages of CXCR5 expressing-cells and intensity of surface expression within each B-cell population. (H) In vitro migration index of total, mature activated, mature MZ-like, precursor MZ-like, switched resting memory and TI B-cells from the blood of classic progressors (5–8 months PI) (n = 6), aviremic slow progressors/elite controllers (EC) (n = 6) and HIV-negative individuals (n = 6) in response to 500 ng/ml CXCL13. The in vitro migration index is defined by the number of cells that have migrated in response to a given chemokine divided by the number of cells that have spontaneously migrated. Data were compared using the Wilcoxon signed rank test and the Mann-Whitney U test for pairwise comparisons of different phases of infection within each group and between the study groups, respectively. Data shown are mean ± SEM. Significance for percentages are expressed by * p < 0.05; ** p < 0.001; *** p < 0.0001 and intensity of surface expression by # p < 0.05; ## p < 0.001; ### p < 0.0001 when compared to HIV-negative individuals. ¤ p < 0.05 for pairwise comparisons of percentages. PI, post-infection; ART, antiretroviral therapy.

**Fig 3:**
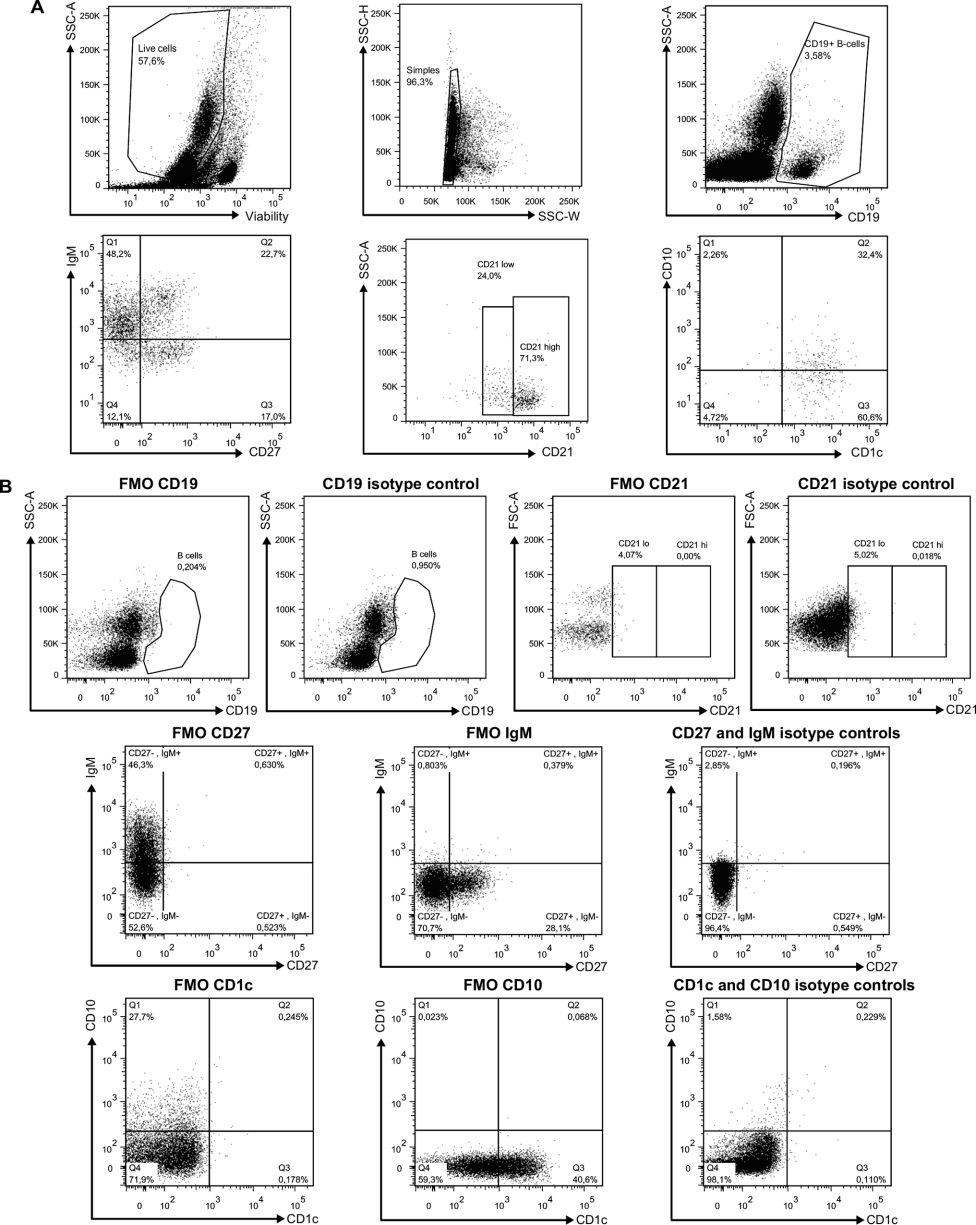
Flow-Cytometry gating strategy for analysis of blood B-cell populations of HIV-1 infected individuals. Representative plot showing gating strategy on 105 live PBMCs. Total CD19+ B-cells were selected based on expression of CD27 and/or IgM, and levels of CD21. CD1c and CD10 expression were used for further characterization of blood MZ and TI B-cell populations. Quadrants were set based on the expression values obtained with fluorescence minus one (FMO) and isotype controls. Mature activated B-cells were defined as CD19+CD27+IgM-CD21loCD1c-CD10-, resting switched memory B-cells were CD19+CD27+IgM-CD21hiCD10-, precursor marginal-zone (MZ)-like B-cells were CD19+CD27+IgM+CD21loCD1c+CD10+, mature MZ-like B-cells were CD19+CD27+IgM+CD21hiCD1c+CD10- and transitional immature (TI) B-cells were CD19+CD27-IgM+CD21hiCD1c-CD10+. The mean events gated were: total B-cells (9320 ± 1750), mature activated (360 ± 67), resting switched memory (632 ± 301), precursor MZ-like (145 ± 36) mature MZ-like (327 ± 233) and TI (944 ± 174).

CXCL13 levels were increased as soon as in the acute phase and throughout the course of infection in all viremic patients (Fig 2A). CXCL13 levels in slow progressors were slightly above those observed in HIV-negative controls (Fig 2A, right panel). Frequencies of cells expressing chemokine receptors (%), as well as surface expression levels (GeoMFI) were assessed based on the strategy of flow-cytometry analysis depicted in Fig 3. When compared to HIV-negative controls, there were significant decreases in frequencies of total as well as mature activated (CD19^+^CD27^+^IgM^-^CD21^lo^) B-cells expressing CXCR5 in the blood of both rapid and classic progressors for most time-points, throughout follow-up and despite therapy. Surface expression levels of CXCR5 were also significantly reduced on these populations in these individuals (Fig 2B and 2C left and middle panels). Interestingly, although slow progressors showed no or slight difference in the percentage of CXCR5 expressing total or mature activated B-cells when compared to HIV-negative controls, CXCR5 surface expression levels were significantly reduced (Fig 2B and 2C right panels). As for resting switched memory B-cells (CD19^+^CD27^+^IgM^-^CD21^hi^), CXCR5 surface expression levels were significantly decreased at most time-points for all HIV-1-infected subjects in comparison to HIV-negative controls (Fig 2D). Interestingly, the frequencies of CXCR5 expressing precursor (CD19^+^CD27^+^IgM^hi^CD21^lo^CD1c^+^CD10^+^) and mature MZ-like (CD19^+^CD27^+^IgM^hi^CD21^hi^CD1c^+^CD10^-^) populations remained unaltered during the course of infection in all HIV-1-infected individuals (Fig 2E and 2F). However, surface expression levels of CXCR5 on mature MZ-like B-cells were significantly lower in all viremic HIV-infected patients when compared to those observed in HIV-negative controls (Fig 2F). The frequencies of CXCR5 expressing TI (CD19^+^CD27^-^IgM^+^CD21^+^CD10^+^) B-cells were lower at the 0–3 month time-point for rapid and classic progressors in comparison to HIV-negative controls (Fig 2G) and no changes were observed regarding surface expression levels (Fig 2G). Overall, the most important difference between HIV-1-uninfected and -infected individuals, regardless of disease progression type, is observed in the more mature B-cell populations, such as mature activated, resting switched memory and mature MZ-like, where cells express significantly less surface CXCR5 than the HIV-1 negative controls, likely reflecting response to the increased CXCL13 plasma levels at the same time-points. Classic progressors and aviremic slow progressors/EC had similar *in vitro* migration indexes in response to CXCL13, with a trend for greater increase for precursor MZ-like B-cells, and for TI of aviremic slow progressors/EC (Fig 2H).

### Longitudinal measurements of CXCL12 plasma levels and CXCR4 expression by blood B-cells of HIV-1-infected individuals with different rates of disease progression

**Fig 4:**
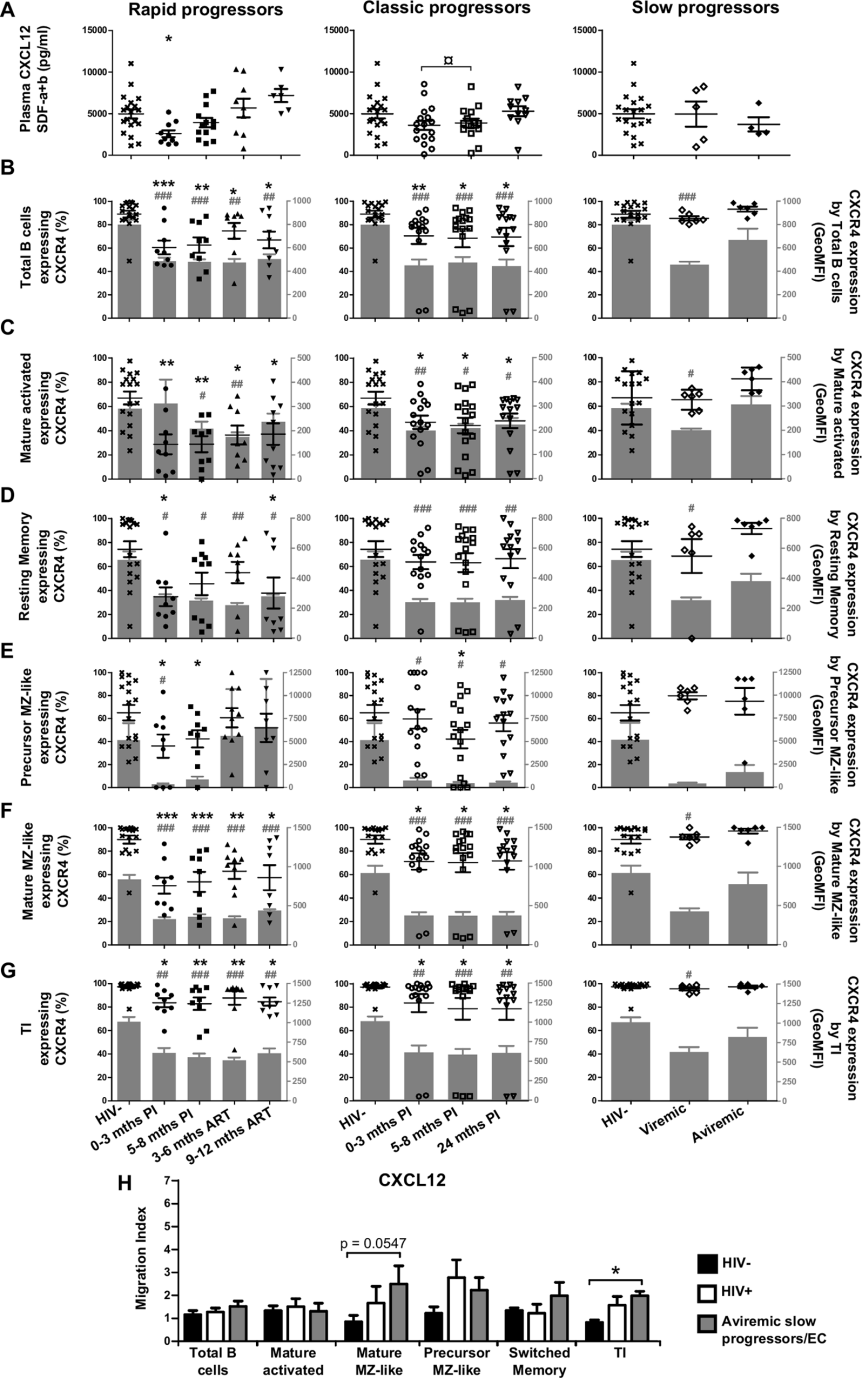
Analysis of plasma CXCL12 levels, CXCR4 expression and migratory potential of by blood B-cells of HIV-infected individuals. (A) Plasma concentrations (pg/ml) of CXCL12 in rapid (left panel), classic (middle panel) and slow progressors (right panel). The same HIV-negative values are used as a control for all three panels. Frequencies of B-cells expressing CXCR4 (left y axis) and levels of CXCR4 surface expression (geometric mean fluorescence intensity -geoMFI) (right y axis) by (B) total, (C) mature activated, (D) resting switched memory, (E) ‘precursor’ marginal zone (MZ)-like, (F) ‘mature’ MZ-like and (G) transitional immature (TI) B-cells of rapid (left panel), classic (middle panel) and slow progressors (right panel). The same HIV-negative values are used as a control for all three panels. Data are expressed as percentages of CXCR4 expressing-cells and intensity of surface expression within each B-cell population. (H) In vitro migration index of total, mature activated, mature MZ-like, precursor MZ-like, switched resting memory and TI B-cells from the blood of classic progressors (5–8 months PI) (n = 6), aviremic slow progressors/elite controllers (EC) (n = 6) and HIV-negative individuals (n = 6) in response to 250 ng/ml CXCL12. The in vitro migration index is defined by the number of cells that have migrated in response to a given chemokine divided by the number of cells that have spontaneously migrated. Data were compared using the Wilcoxon signed rank test and the Mann-Whitney U test for pairwise comparisons of different phases of infection within each group and between the study groups, respectively. Data shown are mean ± SEM. Significance for percentages are expressed by * p < 0.05; ** p < 0.001; *** p < 0.0001 and intensity of surface expression by # p < 0.05; ## p < 0.001; ### p < 0.0001 when compared to HIV-negative individuals. ¤ p < 0.05 for pairwise comparisons of percentages. PI, post-infection; ART, antiretroviral therapy.

CXCL12 levels were decreased in the acute phase of infection for both rapid and classic progressors, and subsequently increased in the chronic phase (Fig 4A left and middle panels). CXCL12 levels in slow progressors were similar to those observed in HIV-negative controls (Fig 4A right panel). CXCR4 was expressed by the majority of blood B-cell populations analyzed. Both the levels of surface expression and frequencies of CXCR4 expressing cells were reduced for all B-cell populations in HIV-1-infected rapid and classic progressors when compared to HIV-negative controls (Fig 4B–4G). As for slow progressors, percentages of CXCR4 expressing B-cells were similar to that of HIV-negative controls (Fig 4B and 4C, right panels). CXCR4 surface expression levels in viremic slow progressors were significantly reduced in most B-cell populations analyzed when compared to aviremic slow progressors/EC and HIV-negative controls (Fig 4B–4G right panels). Overall, the modulation of CXCL12 and CXCR4 was most evident in viremic individuals whereas aviremic slow progressors/EC appeared least affected. Classic progressors and aviremic slow progressors/EC had higher *in vitro* migration indexes in response to CXCL12 for mature MZ-like, precursor MZ-like and TI when compared to HIV-negative controls (Fig 4H).

### Longitudinal measurements of CXCL11 plasma levels and CXCR7 expression by blood B-cells of HIV-1-infected individuals with different rates of disease progression

**Fig 5:**
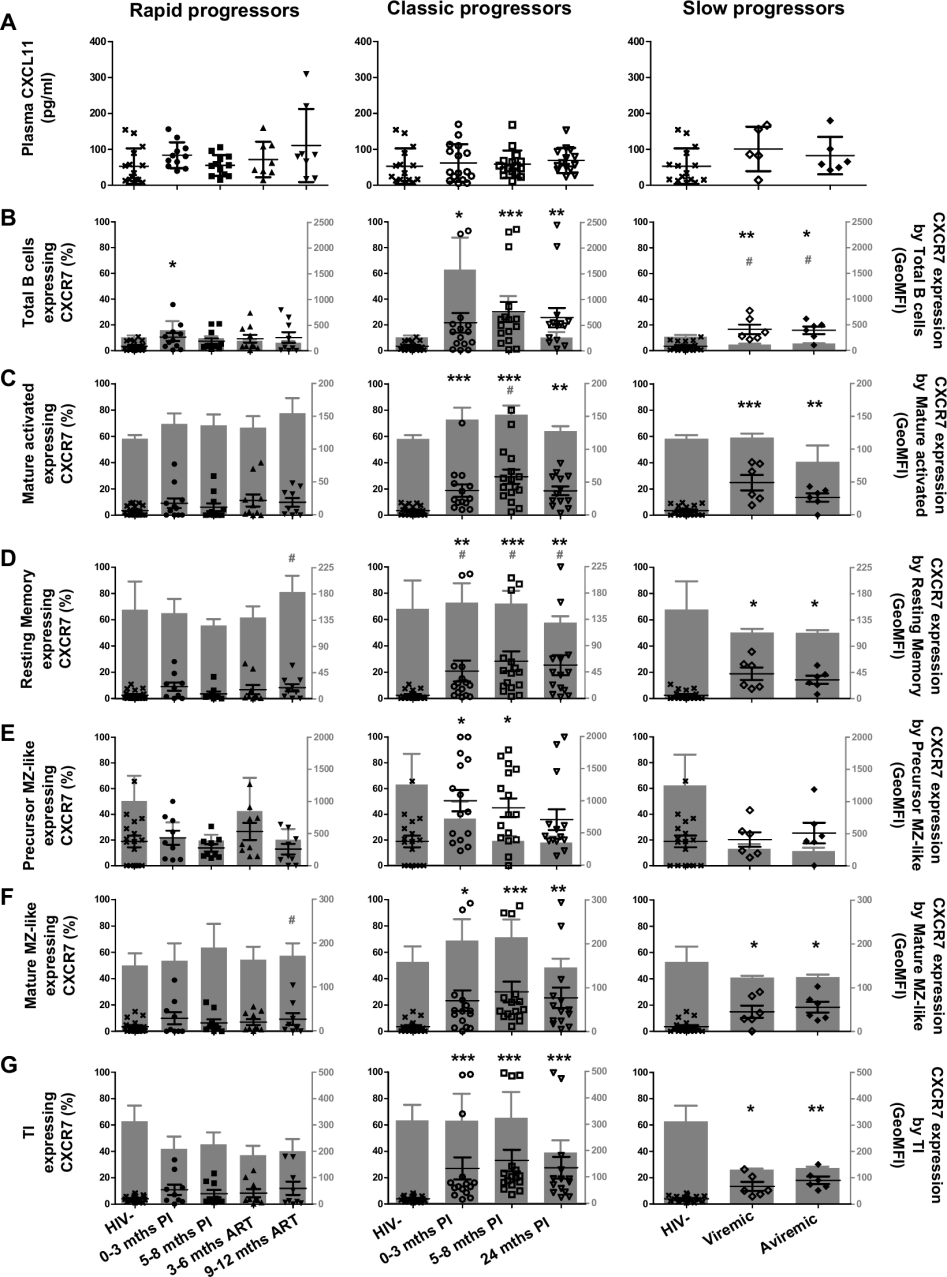
Longitudinal analysis of plasma CXCL11 levels and CXCR7 expression by blood B-cells of HIV-infected individuals. (A) Plasma concentrations (pg/ml) of CXCL11 in rapid (left panel), classic (middle panel) and slow progressors (right panel). The same HIV-negative values are used as a control for all three panels. Frequencies of B-cells expressing CXCR7 (left y axis) and levels of CXCR7 surface expression (geometric mean fluorescence intensity -geoMFI) (right y axis) by (B) total, (C) mature activated, (D) resting switched memory, (E) ‘precursor’ marginal zone (MZ)-like, (F) ‘mature’ MZ-like and (G) transitional immature (TI) B-cells of rapid (left panel), classic (middle panel) and slow progressors (right panel). The same HIV-negative values are used as a control for all three panels. Data are expressed as percentages of CXCR7 expressing-cells and intensity of surface expression within each B-cell population. Plasma concentrations and receptor frequencies were compared using the Wilcoxon signed rank test and the Mann-Whitney U test for pairwise comparison of different phases of infection within each groups and between study groups, respectively. Data shown are mean ± SEM. Significance for percentages are expressed by * p < 0.05; ** p < 0.001; *** p < 0.0001 and intensity of surface expression by # p < 0.05; ## p < 0.001; ### p < 0.0001 when compared to HIV-negative individuals. ¤ p < 0.05 for pairwise comparisons of percentages. PI, postinfection; ART, antiretroviral therapy.

CXCR7, which binds to CXCL12 with higher affinity than CXCR4, also binds to CXCL11 [15]. CXCL11 levels in all HIV-1-infected individuals were similar to those of HIV-negative controls (Fig 5A). CXCR7 was not greatly expressed by total blood B-cells of HIV-negative controls, but its expression seemed to be favoured by the precursor MZ-like population (Fig 5B–5G). In rapid progressors, the levels of surface expression and frequencies of CXCR7 expressing B-cells were not significantly altered when compared to HIV-negative controls (Fig 5B–5G left panels). However, in classic progressors, we found that the percentages of CXCR7 expressing cells were significantly increased when compared to HIV-negative controls at all time-points for total and all B-cell populations analyzed (Fig 5B–5G middle panels). We found no significant changes for CXCR7 surface expression levels except for resting switched memory B-cells (Fig 5D middle panel). Importantly, we found that the frequencies of CXCR7 expressing B-cells were significantly higher in slow progressors when compared to HIV-negative controls, for all populations except precursor MZ-like (Fig 5B–5G, right panels). CXCR7 surface expression levels were significantly lower on total B-cells (Fig 5B right panel), and had a strong tendency to be lower for most populations of these individuals (Fig 5B–5G right panels). CXCL11 was not assessed in our final migration assays but the expression of CXCR7 needs to be taken into consideration when interpreting B-cell responses to CXCL12. The fact that frequencies of CXCR7 expressing B-cells were significantly increased within most populations analyzed in aviremic slow progressors/EC likely reflects an effort to maintain the integrity of the CXCL12-CXCR4 axis in these individuals.

### Longitudinal measurements of CCL20 plasma levels and CCR6 expression by blood B-cells of HIV-1-infected individuals with different rates of disease progression

**Fig 6:**
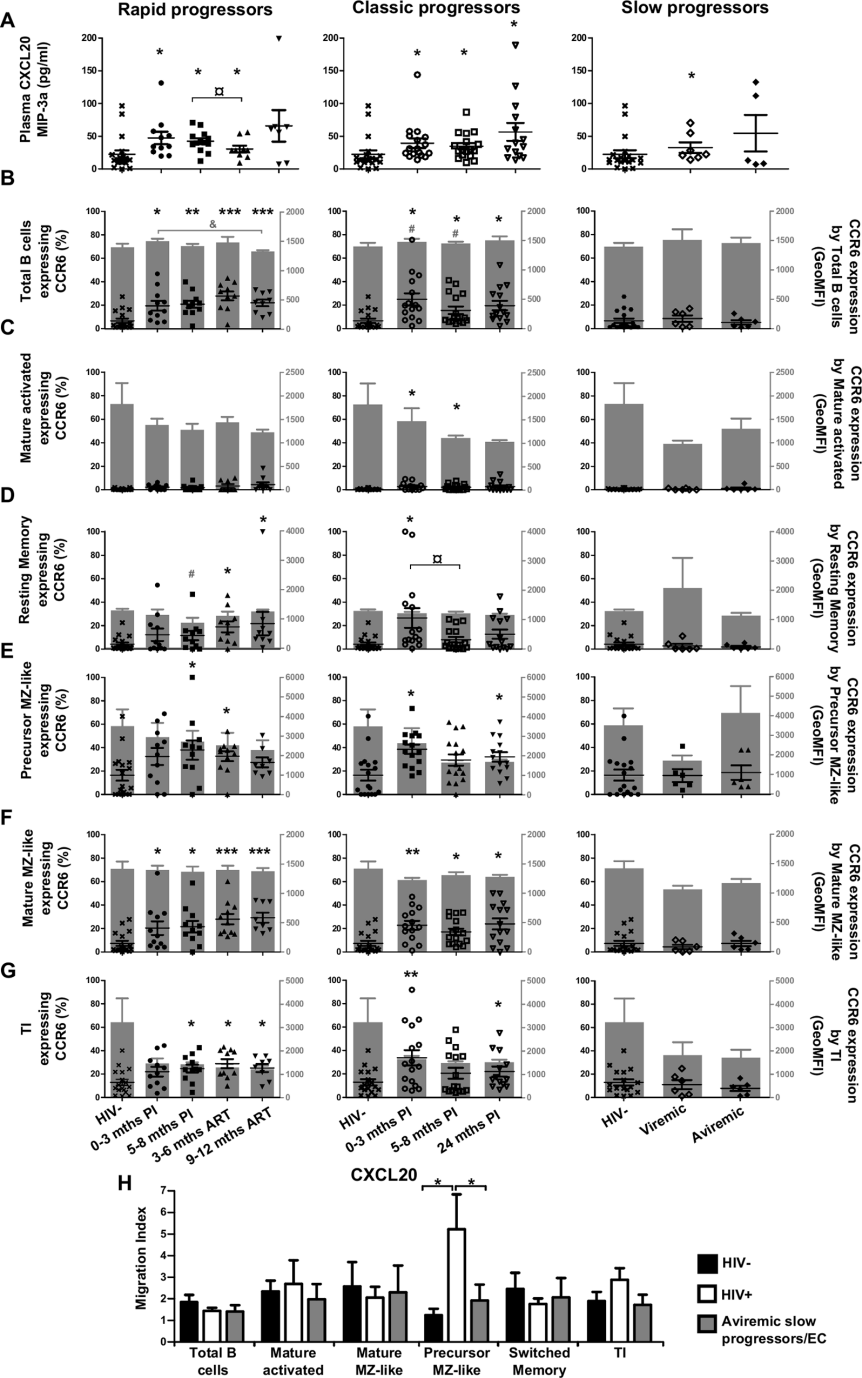
Analysis of plasma CCL20 levels, CCR6 expression and migratory potential by blood B-cells of HIV-infected individuals. (A) Plasma concentrations (pg/ml) of CCL20 in rapid (left panel), classic (middle panel) and slow progressors (right panel). The same HIV-negative values are used as a control for all three panels. Frequencies of B-cells expressing CCR6 (left y axis) and levels of CCR6 surface expression (geometric mean fluorescence intensity -geoMFI) (right y axis) by (B) total, (C) mature activated, (D) resting switched memory, (E) ‘precursor’ marginal zone (MZ)-like, (F) ‘mature’ MZ-like and (G) transitional immature (TI) B-cells by rapid (left panel), classic (middle panel) and slow progressors (right panel). The same HIV-negative values are used as a control for all three panels. Data are expressed as percentages of CCR6 expressing-cells and intensity of surface expression within each B-cell population. (H) In vitro migration index of total, mature activated, mature MZ-like, precursor MZ-like, switched resting memory and TI B-cells from the blood of classic progressors (5–8 months PI) (n = 6), aviremic slow progressors/elite controllers (EC) (n = 6) and HIV-negative individuals (n = 6) in response to 100 ng/ml CCL20. The in vitro migration index is defined by the number of cells that have migrated in response to a given chemokine divided by the number of cells that have spontaneously migrated. Data were compared using the Wilcoxon signed rank test and the Mann-Whitney U test for pairwise comparisons of different phases of infection within each group and between the study groups, respectively. Data shown are mean ± SEM. Significance for percentages are expressed by * p < 0.05; ** p < 0.001; *** p < 0.0001 and intensity of surface expression by # p < 0.05; ## p < 0.001; ### p < 0.0001 when compared to HIV-negative individuals. ¤ p < 0.05 and & p < 0.05 for pairwise comparisons of percentages and intensity of surface expression, respectively. PI, postinfection; ART, antiretroviral therapy.

CCL20 levels were significantly increased at all time-points for all viremic subjects when compared to HIV-negative controls (Fig 6A). CCR6 was expressed by a relatively small percentage of total blood B-cells of HIV-negative individuals, and among the populations analyzed, we found more CCR6 expressing cells within the precursor and mature MZ-like, as well as TI B-cells (Fig 6B–6G). We found that for rapid and classic progressors, the frequencies of CCR6 expressing cells were higher than normal at most time-points, for these and most B-cell populations analyzed (Fig 6B–6G left and middle panels). Levels of CCR6 surface expression were however not greatly affected in these individuals (Fig 6E–6G left panels). We found no significant changes in frequencies of CCR6 expressing cells or levels of surface expression for most B-cell populations in slow progressors when compared to HIV-negative controls (Fig 6B–6G right panels). Overall, we found the greatest modulation of the CCR6-CCL20 axis in rapid and classic progressors, which is consistent with active cellular recruitment towards peripheral and mucosal sites. Upon assessment of B-cell migration potential towards CCL20 (Fig 6H), classic progressors showed the highest migration index for precursor MZ-like B-cells.

### Longitudinal measurements of CCL25 plasma levels and CCR9 expression by blood B-cells of HIV-1-infected individuals with different rates of disease progression

**Fig 7:**
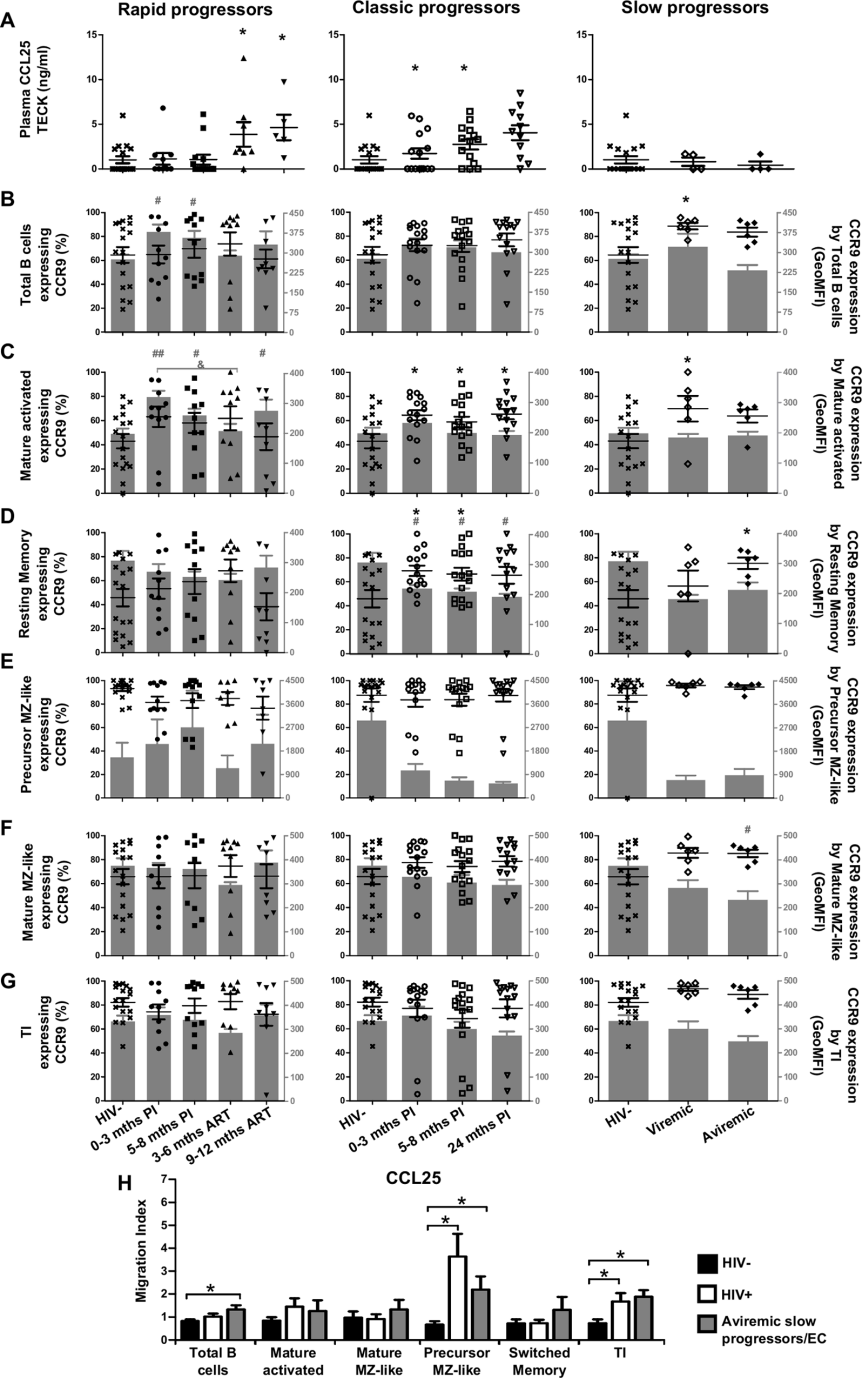
Analysis of plasma CCL25 levels, CCR9 expression and migratory potential by blood B-cells of HIV-infected individuals. (A) Plasma concentrations (pg/ml) of CCL25 in rapid (left panel), classic (middle panel) and slow progressors (right panel). The same HIV-negative values are used as a control for all three panels. Frequencies of B-cells expressing CCR9 (left y axis) and levels of CCR9 surface expression (geometric mean fluorescence intensity -geoMFI) (right y axis) by (B) total, (C) mature activated, (D) resting switched memory, (E) ‘precursor’ marginal zone (MZ)-like, (F) ‘mature’ MZ-like and (G) transitional immature (TI) B-cells by rapid (left panel), classic (middle panel) and slow progressors (right panel). The same HIV-negative values are used as a control for all three panels. Data are expressed as percentages of CCR9 expressing-cells and surface expression within each B-cell population. (H) In vitro migration index of total, mature activated, mature MZ-like, precursor MZ-like, switched resting memory and TI B-cells from the blood of classic progressors (5–8 months PI) (n = 6), aviremic slow progressors/elite controllers (EC) (n = 6) and HIV-negative individuals (n = 6) in response to 100 ng/ml CCL25. The in vitro migration index is defined by the number of cells that have migrated in response to a given chemokine divided by the number of cells that have spontaneously migrated. Data were compared using the Wilcoxon signed rank test and the Mann-Whitney U test for pairwise comparisons of different phases of infection within each group and between the study groups, respectively. Data shown are mean ± SEM. Significance for percentages are expressed by * p < 0.05; ** p < 0.001; *** p < 0.0001 and intensity of surface expression by # p < 0.05; ## p < 0.001; ### p < 0.0001 when compared to HIV-negative individuals. & p < 0.05 for pairwise comparisons of intensity of surface expression. PI, postinfection; ART, antiretroviral therapy.

CCL25 levels were modulated at later time-points following infection, and were increased during ART for rapid progressors, and at 5–8 and 24 month PI for classic progressors, whereas slow progressors showed no difference when compared to HIV-negative controls (Fig 7A). We found a heterogeneous distribution of blood frequencies of CCR9 expressing B-cells in the HIV-negative group, and for most populations analyzed with the exception of precursor MZ-like B-cells, which majority expressed CCR9 (Fig 7B–7G). No significant change in the frequency of CCR9 expressing cells was seen in any of the populations analyzed for rapid progressors when compared to HIV-negative controls, although we found increased CCR9 surface expression levels by total and mature activated cells (Fig 7B–7G left panels). Classic and slow progressors had significantly increased frequencies of CCR9 expressing mature activated and resting switched memory B-cells when compared to HIV-negative controls (Fig 7C and 7D middle and right panels). Moreover, CCR9 surface expression levels tended to be lower on certain B-cell populations in these individuals, especially for switched memory as well as precursor and mature MZ-like B-cells (Fig 7D–7F right panels). Interestingly, precursor MZ-like and TI B-cells presented the greatest migration index in response to CCL25 in classic progressors and aviremic slow progressors/EC when compared to HIV-negative controls (Fig 7H). Overall, precursor MZ-like B-cells mostly express CCR9 and appear to be highly solicited by CCL25.

## Discussion

Consistent with reduced total B-cells and altered population frequencies [6,7], B-tropic chemokine-axes were strongly modulated in the blood of HIV-1-infected rapid and classic progressors. This is likely reflecting cellular drainage to peripheral sites; activation, exhaustion and high turnover; as well as altered regulation of cell influx [4,5,16]. Furthermore, we find several B-cell populations from classic progressors had higher migration indexes than HIV-negative controls. These observations are in line with the overwhelming burden and inflammatory status associated with disease progression as well as the high BLyS/BAFF [6] and B-cell IL-10 expression levels [7] we described for viremic HIV-infected individuals, and consistent with the notion that activated cells are more responsive to *in vitro* chemotaxis [17].

In contrast, we found a modest modulation of chemokine-axes in the blood of aviremic slow progressors/EC, which is consistent with their lower inflammatory, BLyS/BAFF and IL-10 expression profiles [6,7]. Interestingly, for these individuals, most *in vitro* migration patterns in response to B-cell tropic chemokines were comparable and sometimes greater than that observed for classic progressors. As such, B-cells from aviremic slow progressors/EC may present receptors less saturated or desensitized by chemokines and other possible ligands. Nevertheless, the mechanisms responsible for differential migratory capacities are complex and may be modulated *in vivo* and deserve to be further elucidated.

Lymphoid organization is severely altered over the course of HIV-1 infection [14]. Here we show that CXCL13 was elevated in the plasma of HIV-1-infected individuals with detectable viremia, early on and despite successful ART. The most important difference between HIV-1-infected and -uninfected individuals, regardless of disease progression type, was observed in the more mature B-cell populations, where cells expressed significantly less surface CXCR5 than the HIV-negative controls, likely reflecting response to CXCL13 plasma levels at the same time-points, as CXCR5 is known to internalize after contact with its ligand [14]. Modulation of the CXCL13-CXCR5 axis has been previously observed in chronic HIV-1-infected individuals [18] as well as in SIV-infected non-human primates [19] and supports drainage of B-cells to the follicles of lymphoid tissues. This is in agreement with the increased number of follicles as well as B-cell hyperplasia seen in the context of SIV/HIV infections [20] and is reminiscent of that we observed in HIV-transgenic mice [21,22]. Increased CXCL13 is also likely to influence positioning within the lymphoid organ, as well as migration to germinal centers [19]. Studies have shown the importance of Lymphotoxin-α in the organization and maintenance of lymphoid structures, as well as in the modulation of immune responses [23], through a process involving a CXCL13 feedback loop [24]. As such, the aviremic slow progressors/EC studied herein had increased frequencies of MZ-like B-cells expressing Lymphotoxin-α when compared to rapid and classic progressors [7], and this may help explain their lower CXCL13 levels and likely a certain lymphoid homeostasis/integrity.

CXCL12 is constitutively expressed in many organs including the normal intestinal epithelium where it contributes to migration and maintenance of barrier integrity [16]. Furthermore, it has recently been found to be modulated in several inflammatory and autoimmune processes [16]. Consistently, we found that the CXCL12-CXCR4 axis was mostly affected in rapid progressors, who presented disrupted mucosal integrity as indicated by elevated plasma LPS and LBP levels [6]. However, we found no significant change in CXCR7 expression in these individuals. Among its many functions, CXCR7 acts as a scavenger and controls the levels of CXCL12 available for CXCR4. It promotes homeostatic and inflammatory migration as well as anti-apoptotic and survival signalling [15,25]. Although we found little modulation of chemotaxis-axes in aviremic slow progressors/EC, their modulation of CXCR7 expression may be advantageous in preserving a seemingly normal CXCL12-CXCR4 axis, as well as mucosal integrity [6]. Of note, CXCR7 surface expression was higher on ‘precursor’ MZ-like B cells for all subjects including HIV-negative individuals. This is in agreement with a recent publication suggesting CXCR7’s involvement in MZ retention [26]. Furthermore, *cxcr7* has been reported in gene expression array analyses of mouse and human MZ B-cells [27].

HIV-infection majorly involves mucosal sites, both for entry and propagation/perpetuation [28]. We found that CCL20 was increased in plasma early on for rapid and classic progressors, and persisted beyond ART. This is consistent with previous studies showing increased CCL20 in the context of inflammation [29,30]. Moreover in these individuals, the frequencies of CCR6 expressing cells were significantly higher than normal at all time-points, for most populations analyzed, especially precursor and mature MZ-like as well as TI B-cells, which mostly expressed CCR6 in HIV-negative controls. Importantly, we found no significant changes in frequencies of CCR6 expressing cells or levels of surface expression in either total blood B-cells or populations studied for either viremic or aviremic slow progressors/EC when compared to HIV-negative controls. This is suggesting a certain degree of integrity/homeostasis of peripheral mucosal B-cell responses in these individuals. We found classic progressors had the highest migration index for the precursor MZ-like population. These observations suggest high turnover and peripheral drainage of precursor MZ B-cells in the context of HIV-disease progression.

Surprisingly, we found CCL25 was only elevated during the chronic phase or following ART for the rapid and classic progressors. We would have expected this chemokine to be elevated at earlier time-points in order to attract cells to the area most affected by HIV-infection. An important accumulation of B-cells has been shown in the gut of SIV-infected macaques [31,32], it is therefore likely that this phenomenon is true in humans as well. It is possible that gut homing may be affected and/or fulfilled through different mechanisms/migratory axes. Of note, we found that levels of retinoic acid in the plasma of rapid progressors were significantly decreased early on following infection when compared to uninfected donors, and these were increased only following ART [33]. Intriguingly, we noticed that the frequency of CCR9 expressing cells is around 80% for most B-cell populations in the slow progressors in contrast to a broader distribution among the other study groups (Fig 7B–7G). This of course could be in link with the smaller sample size, but could also suggest an advantage and/or consequence of chronicity and slow disease progression. Interestingly, switched memory as well as mature MZ-like B-cells presented a greater migration index in response to CCL25 in aviremic slow progressors/EC when compared to classic progressors and uninfected controls. This is suggesting that the capacity of these populations to migrate to the gut could be favoured in EC. Furthermore, upon assessment of migratory capacity in chronically infected classic progressors, we found that precursor MZ-like presented a greater migration index than uninfected controls, and this was also true for aviremic slow progressors/EC. A similar pattern was found for TI in these study groups. Again, these observations suggest high turnover and peripheral drainage of precursor MZ B-cells in the battle against HIV, and the recruitment of mature MZ-like and switched memory populations might be advantageous to control of disease progression.

BLyS/BAFF is known to highly influence cell fate decision towards the MZ B-cell pool [34,35], and our previous studies suggest the BLyS/BAFF expression profile encountered in HIV-infected progressors vs controllers may impact on MZ-like B-cell population dynamics and activation status [6,7,36]. Despite the fact that precursor MZ-like B-cells represent only a small fraction of circulating B-cells, their increased relative frequencies in the blood of rapid and classic progressors in the context of high BLyS/BAFF, and as shown here preferred recruitment to periphery, may significantly influence disease progression. As to whether the precursor MZ-like B-cell population we characterized is involved in over-representation of polyreactive at the expense of refined antibody responses with potent eradicative properties remains to be explored. Furthermore, MZ B-cells set the stage for germinal center reactions [37], and their alteration is likely to impede on high affinity B-cell immunity. Although, the frequency of precursor MZ-like B-cells is unaltered in the blood of aviremic slow progressors/EC, which is consistent with a seemingly preserved BLyS/BAFF and IL-10 expression profile [6,7], they present a high migratory potential in response to chemokines *in vitro*, even though their ex vivo assessment of chemokine receptor surface expression reveals little significant modulation, at the exception of CXCR7 as discussed above. This is suggesting that “regulated” attraction of these cells in a non-inflammatory environment [6,7] could be beneficial to the battle and even control of HIV.

## Conclusion

The mechanisms involved in the control of HIV-1 disease progression and maintenance of a certain degree of immune integrity are likely to require regulated trafficking, and possibly recruitment of populations such as MZ-like B-cells to peripheral and mucosal sites. Efficiently soliciting such populations may be bared in mind in the design of vaccine strategies aiming at generating both first-line/innate and adaptive protective responses.
